# Clinical utility of a pediatric hand exoskeleton: identifying users, practicability, and acceptance, and recommendations for design improvement

**DOI:** 10.1186/s12984-022-00994-9

**Published:** 2022-02-11

**Authors:** Jan Lieber, Jan Dittli, Olivier Lambercy, Roger Gassert, Andreas Meyer-Heim, Hubertus J. A. van Hedel

**Affiliations:** 1 Swiss Children’s Rehab – Research Department, University Children’s Hospital Zurich, Mühlebergstrasse 104, CH-8910 Affoltern am Albis, Switzerland; 2 Children’s Research Center, University Children’s Hospital Zurich, University of Zurich, Steinwiesstrasse 75, 8032 Zurich, Switzerland; 3 Rehabilitation Engineering Laboratory, Department of Health Sciences and Technology, ETH Zurich, 8008 Zurich, Switzerland

**Keywords:** Wearable robots, Pediatric neurorehabilitation, Appropriateness, Practicability, Acceptability, Disability, Hand function, Bimanual performance

## Abstract

**Background:**

Children and adolescents with upper limb impairments can experience limited bimanual performance reducing daily-life independence. We have developed a fully wearable pediatric hand exoskeleton (PEXO) to train or compensate for impaired hand function. In this study, we investigated its appropriateness, practicability, and acceptability.

**Methods:**

Children and adolescents aged 6–18 years with functional limitations in at least one hand due to a neurological cause were selected for this cross-sectional evaluation. We characterized participants by various clinical tests and quantified bimanual performance with the Assisting Hand Assessment (AHA). We identified children whose AHA scaled score increased by ≥ 7 points when using the hand exoskeleton and determined clinical predictors to investigate appropriateness. The time needed to don each component and the number of technical issues were recorded to evaluate practicability. For acceptability, the experiences of the patients and the therapist with PEXO were evaluated. We further noted any adverse events.

**Results:**

Eleven children (median age 11.4 years) agreed to participate, but data was available for nine participants. The median AHA scaled score was higher with PEXO (68; IQR: 59.5–83) than without (55; IQR: 37.5–80.5; *p* = 0.035). The Box and Block test, the Selective Control of the Upper Extremity Scale, and finger extensor muscle strength could differentiate well between those participants who improved in AHA scaled scores by ≥ 7 points and those who did not (sensitivity and specificity varied between 0.75 and 1.00). The median times needed to don the back module, the glove, and the hand module were 62, 150, and 160 s, respectively, but all participants needed assistance. The most critical failures were the robustness of the transmission system, the electronics, and the attachment system. Acceptance was generally high, particularly in participants who improved bimanual performance with PEXO. Five participants experienced some pressure points. No adverse events occurred.

**Conclusions:**

PEXO is a safe exoskeleton that can improve bimanual hand performance in young patients with minimal hand function. PEXO receives high acceptance. We formulated recommendations to improve technical issues and the donning before such exoskeletons can be used under daily-life conditions for therapy or as an assistive device.

*Trial registration* Not appropriate

**Supplementary Information:**

The online version contains supplementary material available at 10.1186/s12984-022-00994-9.

## Background

Many relevant daily-life tasks require the use of both hands. Patients with upper limb impairments, including children and adolescents, can show limitations in independence in daily activities. Several therapy concepts have been developed to improve upper limb impairments, including (modified) Constrained Induced Movement Therapy or Bimanual Intensive Therapy. Robot-assisted training can complement such interventions by providing repetitive goal-directed yet engaging movements [1]. These three forms of therapy have been investigated relatively frequently in children with cerebral palsy (CP) [2]. A recent systematic review concluded that the two forms of conventional therapy effectively improve motor function in children with CP [3]. Evidence for the effectiveness of robot-assisted therapy is emerging [3].

In patients with sensorimotor impairments of the hand due to a neurological lesion, fully wearable robotic hand exoskeletons bear the potential to support task-oriented training in the clinic or at home (i.e., therapy robot), or compensate for the loss of function and assist daily-life activities (i.e., assistive technology) [4]. Soft hand exoskeletons are rapidly emerging due to their inherent safety, less complex design, and increased potential for portability and efficacy [4]. In 2018, the authors of two reviews identified 44 [4] and 45 [5] unique devices, and this number is increasing as shown by more recent publications (e.g., [6–8]). However, only a few publications have focused on developing such technologies for children, which entails specific challenges such as accounting for children’s growth in the sizing of dedicated devices or making the device intuitive and easy to use [9]. For example, one group developed an exoskeleton for the thumb that can actuate the carpometacarpal and metacarpophalangeal joints through ranges of motion required for activities of daily living [10]. Another group developed a finger exoskeleton that assists finger flexion and extension but did not include the thumb [11]. However, various functional tasks require the inclusion of both finger and thumb movements, highlighting the need for devices that assist full hand grasping. No results from studies applying such technology in children have been published, despite these studies being crucial for design adoption by the users [9].

To answer these needs, we developed the pediatric hand exoskeleton (PEXO) [12]. In a previous study, we presented the requirements and design modifications for adapting an adult hand exoskeleton [13] to the unique needs of children with neuromotor impairments, and we made a preliminary validation with a 6-year-old child with stroke [12]. The current paper builds on this work and more extensively evaluates the clinical utility of the current prototype of PEXO in a larger group of patients.

In line with Smart [14], we understand clinical utility as a multi-dimensional model that outlines four aspects in practitioners' and patients' judgments: appropriateness, practicability, acceptability, and accessibility. In this study, we evaluated three of these aspects: (i) appropriateness: i.e., actual effectiveness but also relevance, including how meaningful the intervention could be in the broader context of clinical decision-making; (ii) practicability, concerning the functionality and suitability of robotic devices for clinical applications; and (iii) acceptability by patients and therapists to determine whether there are concerns that might affect treatment or practice. We did not investigate accessibility, i.e., costs and cost-effectiveness or availability of the technology, as PEXO is still a research prototype.

The specific research questions were: (i) Appropriateness: can we identify children with upper limb impairments that can improve hand capacity and particularly bimanual performance when using PEXO? Based on our clinical experience, we hypothesized that children with little hand function but good proximal arm muscles can benefit from PEXO. Furthermore, we investigated whether children with upper limb impairments can familiarize themselves with the use of PEXO within a reasonable time. (ii) Practicability: can patients put PEXO on independently, or how long does it take to don PEXO? We also wanted to identify any technical issues during training sessions to improve the design of PEXO further and reported safety issues. (iii) Finally, we investigated the acceptability of PEXO prototypes by asking participants and the supervising therapist various questions concerning the advantages and disadvantages of PEXO. By combining the insights regarding these dimensions of clinical utility, we formulate recommendations to improve PEXO and pediatric hand exoskeletons in general and pave the way for their successful clinical application.

## Methods

### Participants

We included children and adolescents with brain or peripheral nerve damage resulting in functional limitations of at least one hand. Both in- and out-patients, between 6 and 18 years of age, were recruited at the Swiss Children's Rehab clinic. The participants should be able to sit for an hour and understand the tasks of the study protocol. Children or adolescents who were not able to actively flex either their shoulder or their elbow against gravity (manual muscle testing (MMT) score < 3) [15, 16] were excluded from the study. All participants and their legal guardians provided verbal consent to participate in the study: parents and adolescents aged 14 years and older provided written consent.

Age, sex, most affected hand, dominant hand, and handedness were recorded to characterize the participants. We also noted whether the children had received Botox injections in the upper limbs during the six months before the study. Participants were characterised according to six standard clinical assessments comprising the Manual Ability Classification System (MACS), MMT, Selective Control of the Upper Extremity Scale (SCUES), Hypertonia Assessment Tool (HAT), modified Ashworth Scale (MAS), and Functional Independence Measure for Children (WeeFIM). Trained therapists performed the clinical assessments, except for the WeeFIM, which was scored by trained and certified nurses in the center.

The MACS reliably classifies whether and how children handle objects in everyday life. Classifications vary between level I, where children handle objects effortlessly and successfully, and level V, where children do not handle objects at all [17].

MMT was applied to rate upper extremity muscle strength from 0, i.e., no contraction visible, to 5, i.e., movement over the full range of motion against gravity and severe resistance [15, 16]. If the patient can perform the movement against gravity covering the whole range of motion, the MMT is 3. The test protocol included standardized starting positions, a demonstration of the test by the therapist, and the active execution of the movement by the participant. Shoulder and elbow flexion as well as wrist and finger extension were tested.

The SCUES measures upper limb selective voluntary motor control [18], which is defined as the ability 'to selectively activate muscles independently of each other in the context of the requirement for voluntary movement or posture' [19]. Shoulder abduction and adduction, elbow flexion and extension, pro- and supination, wrist flexion and extension, and finger flexion and extension are tested. Each movement is scored on an ordinal scale from 0, indicating no selective motor control, to 3, reflecting normal selective motor control.

The HAT differentiates between the hypertonia categories spasticity, dystonia, and rigidity (or mixed) and consists of seven items [20]. Items 1, 2, and 6 indicate dystonia, 3 and 4 spasticity, and 5 and 7 rigidity. Each limb is scored separately.

Spasticity severity was measured with the MAS that scores the speed-dependent resistance of moving a joint [16]. In this study, the therapist assessed the MAS of the wrist and finger joints by first moving the joint covering the full passive range of motion at a slow pace, followed by a faster movement. The ordinal scale varies from 0 (i.e., no resistance during passive movement) to 4 (i.e., the affected section is rigid in flexion or extension).

The WeeFIM is a valid and reliable instrument assessing the degree of independence on a seven-level scale [21]. The functional assessment includes 18 items covering self-care, mobility, and cognition. The participants were characterized with the WeeFIM total and particularly the self-care score, as the latter contains items reflecting upper limb use in daily activities.

### Assistive hand exoskeleton PEXO

**Fig. 1:**
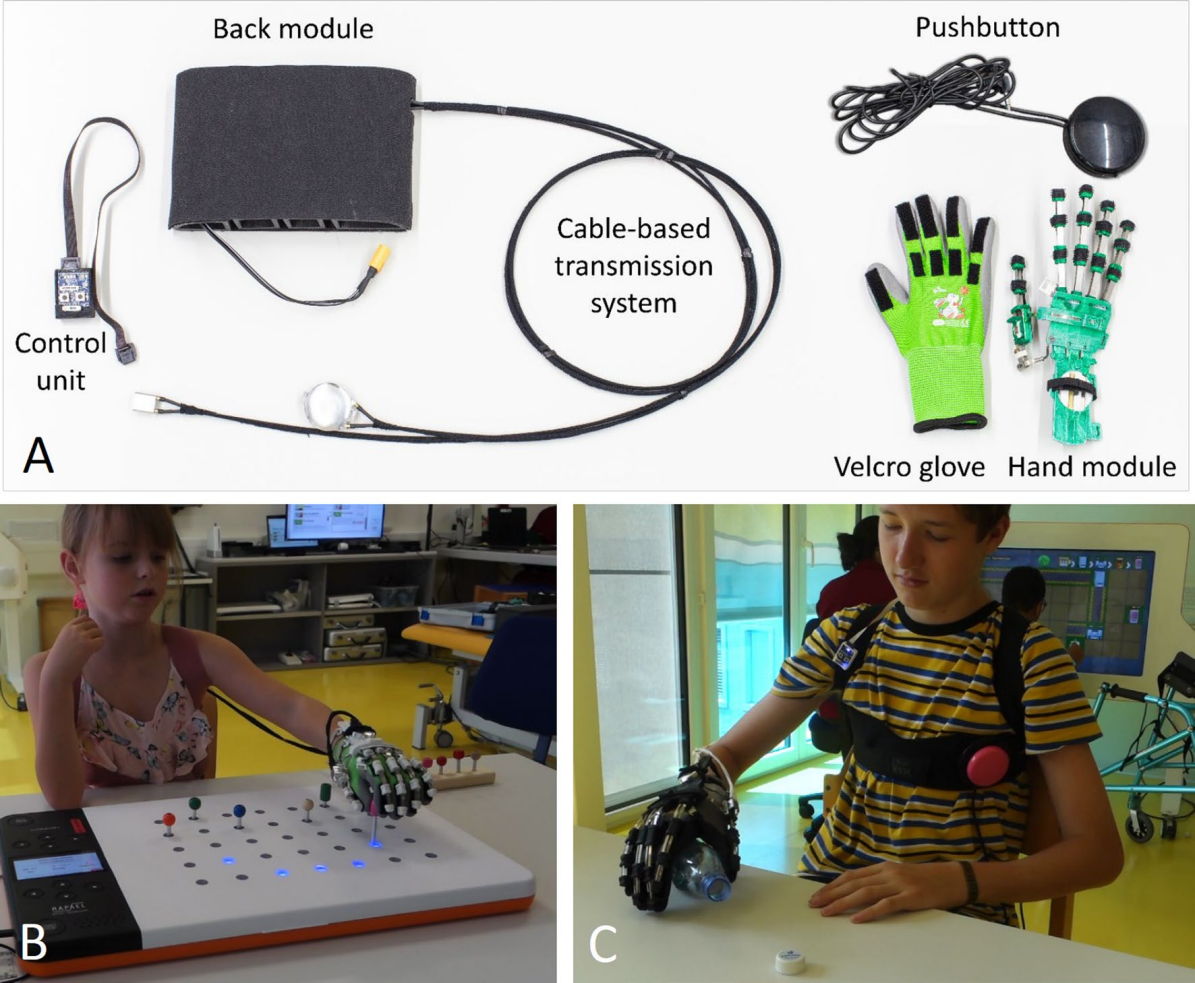
Overview of PEXO and participants performing different tasks with PEXO. **A** PEXO consists of a hand module, a Velcro glove, and a back module containing motors, electronics, and the battery. The power of the motors is transmitted to the hand module via cable-based transmission. Large pushbuttons or a control unit can be used to trigger the opening and closing of PEXO. **B** ID05, female, 7.5 years old, 6 months after being diagnosed with rhabdomyolysis, performing the Shape Completion task with the Smart Pegboard from Neofect. **C** ID03, male, 15.7 years, 2 months after stroke, opening a bottle, as part of the Assisting Hand Assessment, while PEXO assisted in holding the bottle. We received permission from the children and parents to present these pictures

The detailed design of the pediatric assistive hand exoskeleton PEXO was presented previously [12], and an overview of PEXO components is shown in Fig. 1A. In short, PEXO assists full-hand grasping in children with neuromotor impairment by actively supporting flexion and extension of the four fingers (index, middle, ring, and little finger) combined and the thumb separately, using a soft three-layered spring blade mechanism [22]. The thumb of the exoskeleton can be moved to opposition using a passive slider, allowing the users to perform different grasp types relevant for activities of daily living (e.g., power grasp, precision pinch, and lateral grasp). The hand exoskeleton provides sufficient force to grasp objects weighing up to 0.5 kg and closes and opens within 1 s. PEXO consists of a hand module (i.e., the actual exoskeleton) and a back module. The sleek hand module (weight < 105 g, maximum 1.5 cm added height on the back of the hand) is donned on a user's hand using a Velcro glove to fixate the exoskeleton on the fingers. Two straps around the wrist and one strap around the palm securely fix the exoskeleton. The back module (weight 492 g) contains the electronics, motors, and battery to power the hand module via a cable-based transmission system [23]. This design reduces the weight carried on the hand. The entire hand exoskeleton system is fully wearable since the back module can be worn as a backpack or attached to a wheelchair, allowing the user to move around freely (see also Fig. 1B, C). While the hand module of PEXO was explicitly optimized for the application in children in terms of size, weight, design, and functionality [12], the back module remained unchanged from the prior developed RELab tenoexo for adults with neurological hand impairment after stroke or spinal cord injury [13]. This commonality, combined with the possibility of detaching the transmission system from the hand module, allowed for using hand modules of different sizes with only a single back module. Hand modules were prepared in three different sizes for the left and right hand, covering the hand sizes of children aged 6 years, 7 to 8 years, and 9 to 12 years based on anthropometric data. The hand module of the adult RELab tenoexo was used by adolescents between 13 and 18 years of age. Large-diameter pushbuttons were used in this study to trigger the opening and closing movement of PEXO. An additional control unit allowed therapists or other caregivers to adjust the supporting force exerted by the hand exoskeleton.

### Measurement procedures and assessments

The measurements took about two hours and were paused for a break to avoid fatigue of the participants. An experienced occupational therapist (JL) and a research engineer (JD) conducted the measurements. The order of the tests and the instructions were standardized.

First, the patient descriptors (MMT, HAT, MAS, and SCUES) were assessed (without PEXO). To determine the most appropriate PEXO size, participants had to place their hands on wooden stencils, which were created in accordance with the available hand module sizes based on age-appropriate standard anthropometry data of children. Subsequently, the participants were asked to put on the back module, the glove, and PEXO hand module as independently as possible. We recorded the time needed and whether the children needed assistance to put the separate components on. Next, the participants chose a location on the table or the body (e.g., see Fig. 1C) that was easy for them to reach, where the pushbutton to close and open PEXO was placed. Then, while wearing PEXO, the participants performed a standardized assessment with the Smart Pegboard (Neofect, Munich, Germany). This instrumented pegboard is usually used therapeutically to practice reaching, grasping, and transporting movements and fine motor skills. The pegboard includes animated games on an electronic perforated plate with light signals (Fig. 1B). Patients have to insert pegs, which can be of different dimensions, in the holes that are illuminated. For this study, we used pegs (dimensions: length 4 cm, diameter 5 mm) with a knob (diameter 10 mm) on top, allowing a lateral or tip pinch. The time needed by the participant to insert a maximum of eleven pegs was measured, with the maximum test duration being set to 120 s. The number of pegs positioned in the appropriate hole [x/11] was recorded if the participants could not insert all pegs within 120 s. Afterward, the participants had the opportunity to practice the use of PEXO on the pegboard. The practice time was recorded. Then, the pegboard assessment was repeated, once with and once without PEXO.

The grip strength and lateral pinch strength were measured with and without PEXO using the Jamar dynamometer and the finger closure gauge [15, 16, 24]. Reference data for typically developing children and adolescents are available for comparison [15, 16, 24].

We then investigated whether participants could perform various hand movements (i.e., lateral pinch, tip pinch, and fist closure). When assessing the ability to perform various hand movements, the child manually repositioned the PEXO thumb if possible. For those children who were unable to do so, the therapists assisted the child. Furthermore, they performed two functional assessments, the Assisting Hand Assessment (AHA) and the Box and Block Test (BBT), with and without PEXO. The kids-AHA is a test procedure for children between 18 months and 12 years of age and assesses how effectively a child uses its impaired upper extremity (assisting hand) in bimanual tasks. For the analysis, the participant is videotaped in a play situation. Afterward, a trained and certified occupational therapist assesses the spontaneous use of the assisting hand for 20 items. Each item is scored on a scale from 1 to 4 (1—does not do, 2—ineffective, 3—somewhat effective, and 4—effective). Rated items are, for example, whether participants initiate the use of the assisting hand themselves, open a bottle (Fig. 1C), stabilize objects or whether they reach for objects with the assisting hand [25]. The AHA provides raw scores but also scaled scores (0–100), which are derived from Rasch-analysis and are interval-scaled.

The BBT is a capacity test measuring unimanual gross dexterity of the arm and hand. Within 60 seconds, the participants need to move as many blocks as possible from one compartment of the box to the other. Age-appropriate norm values exist for children and adolescents [26, 27].

In line with the International Classification of Functioning, Disability, and Health-Children and Youth Version (ICF-CY), strength as assessed by the Jamar dynamometer is a body function, while the pegboard and the BBT are capacity measures (activity domain), and the kids-AHA is a performance measure (activity domain) [28, 29].

After completing the tests, the therapist supported the participants in doffing PEXO. The participants were asked to rate six statements concerning the training with PEXO on a Likert Scale from 1 (not at all) to 5 (very much). The statements are listed in Table 3 (P1 to P6). Additionally, the participants were questioned regarding pressure points while wearing PEXO, potentially leading to discomfort. If the participants experienced discomfort, these areas were located and the participants were asked to rate the intensity of the caused pain on a Visual Analogue Scale from 0 (no pain) to 10 (worst imaginable pain) [30]. Finally, the participants were asked to give feedback on what they liked and disliked about the therapy with PEXO.

The therapist filled in a custom-made questionnaire consisting of five statements (T1 to T5 in Table 3) and answered three open questions “If the child was not able to perform a goal-oriented training, please specify why this was not possible”, “What was your general impression of training with PEXO for this specific child?”, and “Were there any technical problems? If yes, please describe them in detail and indicate their numbers.” We rated the technical errors by their number of occurrences and severity, comparable to a retrospective failure mode, effect, and criticality analysis (FMECA) [31, 32]. The following severity levels were defined:

1. Negligible issue not influencing performance or functionality.
2. Marginal issue allowing successful task completion, leading to a small delay (< 1 min) and/or requiring additional action/adjustment by the user.
3. Issue allowing successful task completion, leading to a major delay (> 1 min) and/or requiring support from a caregiver.
4. Critical issue requiring intervention by the study coordinator to avoid potential harm to the participants and/or preventing task completion due to total failure of the device requiring technical maintenance

Finally, we noted any PEXO-related adverse events.

### Outcomes and statistical analyses

*Appropriateness* We quantified bimanual hand performance with the AHA scaled score and hand capacity with the BBT. Due to the small sample size, the non-parametric Wilcoxon signed-rank test was performed to determine differences between the conditions with versus without PEXO. The Z-statistic value of the Wilcoxon signed-rank test and the *p*-value were reported.

As a first step in identifying children who could improve bimanual performance and unimanual hand capacity when using PEXO, the non-parametric Spearman's correlations (ρ) were calculated between the differences in AHA scaled scores (i.e., with PEXO minus without PEXO) and various patient characteristics and functional measures. We interpreted the magnitude of the correlation coefficients as follows: 0–0.25 (no or little relationship), 0.25–0.50 (fair degree), 0.50–0.75 (moderate to good relationship), 0.75–1.00 (very good to excellent).

In addition to the correlation analyses, we calculated a dichotomous variable indicating an improvement in bimanual performance yes/no. Based on the standard error of measurement calculated for the intra-rater reliability (raw score: 1.2 points), we estimated the smallest detectable change (2.77 × 1.2 = 3.3) and made a conservative estimation of the smallest detectable change for the scaled scores (i.e., 7 points) using transformation curves published by the authors of the AHA [33], i.e., we interpreted an improvement of 7 points or more when wearing PEXO as a conservative estimate of improved bimanual performance. To identify characteristics and functional measures that differed between the children who could improve bimanual hand performance when wearing PEXO or not, chi-square tests were used to determine differences in dichotomous measures and Wilcoxon signed-rank test to determine differences in ordinal or interval-scaled measures. Furthermore, Receiver Operating Characteristics (ROC) analyses were performed to determine the level of sensitivity and specificity with which the ordinal and interval-scaled measures could distinguish between participants who performed better when wearing PEXO (≥ 7 points improvement in scaled AHA scores) and those who did not.

To investigate familiarization with using PEXO, data from the Smart Pegboard was used (number of correct placements from 11 pegs and time needed to accomplish the task). Differences in the pegboard scores were compared between pre- to post-practice time. The post-practice conditions with versus without PEXO were further compared. While α was generally set at 0.05 for all comparisons, we set it at 0.025 for these multiple comparisons.

*Practicability* Time needed for donning the components of PEXO and whether this could be done independently by the participant. For the time needed to don the back module, the glove, and the hand module, the median and interquartile range (IQR) were calculated and the minimum and maximal values were reported.

Furthermore, the number and nature of technical and safety issues were described.

*Acceptability* The descriptive values of the Likert scores that participants and the therapist provided to the various questions were reported.

## Results

The characteristics of the participants can be found in Table 1. The median age was 11.4 years (IQR 9.4–16.1 years). None of the patients had received Botox during the past months. ID4 was excluded from the data analysis as the measurement protocol could not be completed due to a malfunction of PEXO electronics, preventing the actuation of PEXO. In ID2, a cable of the transmission system ruptured during the measurements before the AHA and BBT could be completed with PEXO (i.e., there are no data available for AHA, BBT, lateral pinch strength, and grip strength with PEXO).

**Table 1:** Characteristics of the participants

ID	Sex	Age [years]	Hand			Diagnosis	Time since lesion	HAT	MACS	WeeFIM		MAS [x/4]		MMT [x(5]				SCUES	PEXO size^*^
			MA	Dom	Handed		[months]			SC	Total	Wrist	Finger	Sh	Elbow	Wrist	Finger	[x/15]	
1	Female	17.7	Left	Right	Right	Ependymoma	90.0	Spas	III	49	118	1 +	1 +	3	4	1	1	5	3
2	Male	10.3	Right	Left	n.a	CP unilat.spast	n.a	Spas	III	^#^	^#^	0	0	4	3	3	3	11	2
3	Male	15.7	Right	left	Left	Stroke	2.0		I	55	118	0	0	4	4	4	4	12	4
4	Female	12.0	Left	Right	Right	TBI	2.0		I	54	112								3
5	Female	7.5	Left	Right	Right	Rhabdomyolysis	6.0		I	38	86	0	0	4	4	4	4	15	2
6	Male	8.0	Right	left	Right	Stroke	2.5	SpasDys	IV	23	57	0	0	3	3	3	3	11	1
7	Male	11.4	Left	Right	Right	Stroke	2.0	Spas	IV	33	78	1	1	2	4	3	0	4	3
8	Female	9.4	Left	left	Right	Neuropathy	97.0		II	24	56	0	0	2	3	3	3	12	2
9	Female	10.7	Left	Right	Right	TBI	114.0	Spas	IV	^#^	^#^	0	1 +	3	4	3	0	5	2
10	Female	16.6	Left	Right	Right	Stroke	185.0	Spas	III	43	92	1	1 +	3	4	1	1	4	3
11	Male	16.1	Right	Left	n.a	CP unilat.spast	n.a	Spas	III	40	81	1 +	1 +	3	3	2	2	6	3

n.a., not appropriate, since time since lesion and handedness were noted for patients with an acquired and not a congenital lesion. #, not available for out-patients. *, size 1: 6 years old, 2: 7–8 years old, 3: 9–12 years old, 4: tenoexo; As PEXO showed a technical failure early during the measurements of ID4, we did not assess the functional tests. CP unilat.spast., unilateral spastic Cerebral Palsy; TBI, traumatic brain injury; HAT, Hypertonia Assessment Tool; MACS, Manual Ability Classification System; WeeFIM, Functional Independence Measure for children; SC, self-care; MAS, Modified Ashworth Scale; MMT, Manual Muscle Test; SCUES, Selective Control of the Upper Extremity Scale; Sh., shoulder

### Appropriateness

**Fig. 2:**
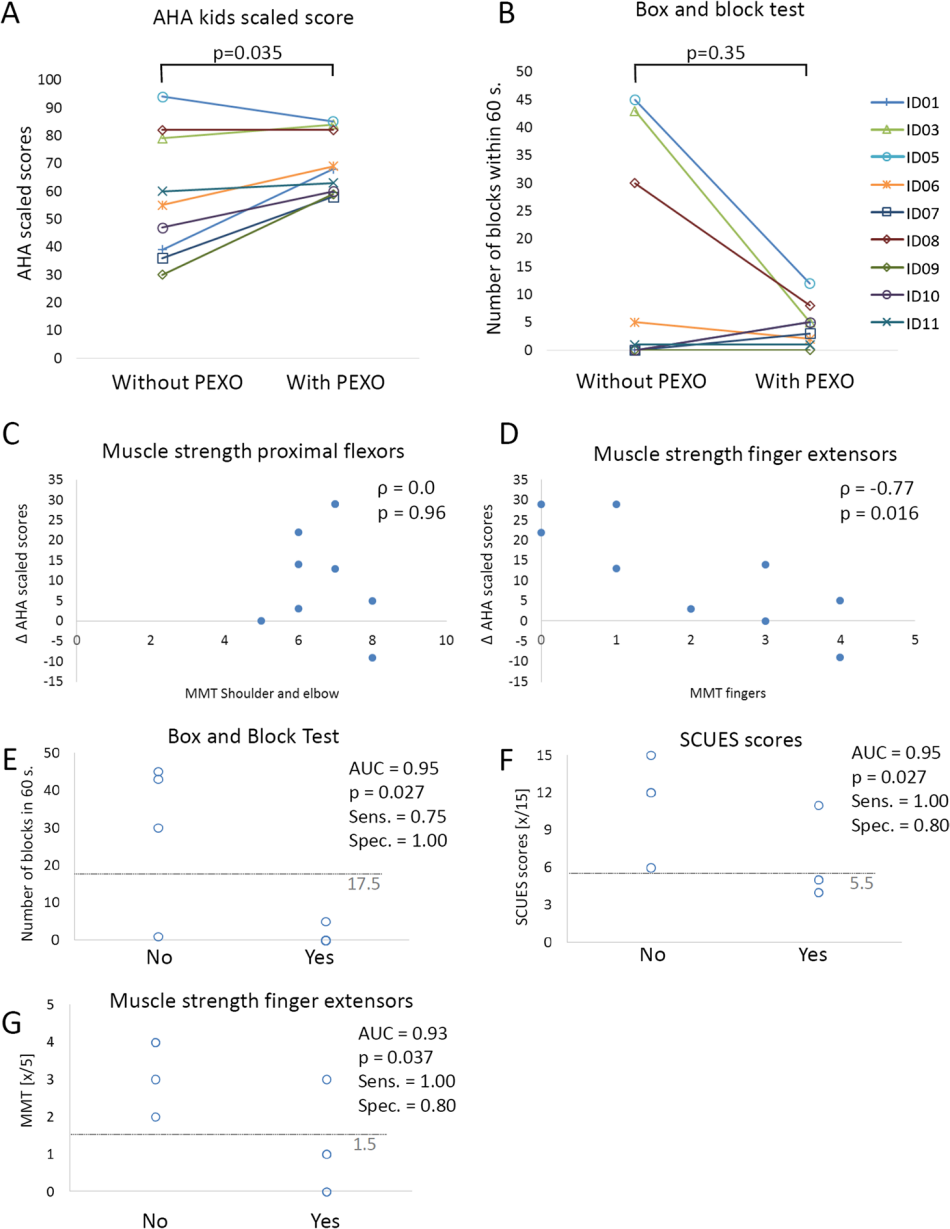
Appropriateness results of PEXO. **A** AHA scaled scores and **B** Box and Block Test scores between conditions without versus with PEXO. Scatterplots show the relationship between the differences (∆) in AHA scaled scores (condition with PEXO minus without) versus **C** the sum of the muscle strength values of the shoulder and elbow flexor, and **D** of the finger extensors. Also shown are results from the Receiver Operating Characteristics analyses. Scatterplots show the cut-off values of the **E** Box and Block Test, **F** the Selective Control of the Upper Extremity Scale (SCUES) scores, and the **G** Manual Muscle Test (MMT) of the finger extensors that could differentiate between participants who could improve bimanual performance when wearing PEXO. The dashed line presents the cut-off value where the maximal combined sensitivity (Sens.) and specificity (Spec.) were found (using the Youden Index). AUC, Area Under the Curve

Differences in AHA scaled scores with versus without PEXO are shown in Fig. 2A. Without PEXO, the median AHA scaled score was 55 (IQR: 37.5–80.5). With PEXO, the median score was significantly higher: 68 (IQR: 59.5–83; Z = − 2.1, *p* = 0.035). The figure shows that participants with lower AHA scaled scores performed better with PEXO. Indeed, a strong negative correlation was found when correlating the AHA scaled score (without PEXO) with the differences (i.e., with minus without PEXO) in AHA scaled score (ρ = − 0.94, *p* < 0.001).

The BBT did not differ significantly between the conditions (without PEXO: median 3 blocks (IQR: 0–33.3); with PEXO: 5 blocks (IQR: 1.5–6.5; Z = − 0.93; *p* = 0.35); Fig. 2B). Participants with high BBT scores performed worse with PEXO than without. Three participants (ID1, ID7, and ID10), who could not transport a single block without PEXO, could move 5, 3, and 5 blocks, respectively, with PEXO. ID9 was not able to transport any block with or without PEXO.

The MACS correlated well with the difference in AHA scaled scores (ρ = 0.76, *p* = 0.018), as did the SCUES score of the more affected upper limb (ρ = − 0.72, *p* = 0.029). Most of the participants had good muscle strength values (MMT ≥ 3) for the shoulder and elbow flexors and wrist extensors (Table 1). Correlations between the difference in AHA scaled scores and MMT scores were at most fair (shoulder: ρ = − 0.26, *p* = 0.50; elbow: ρ = 0.37, *p* = 0.33; wrist: ρ = -0.42, *p* = 0.26), also when combining proximal muscle strength values, i.e., shoulder and elbow scores (Fig. 2C). Only the finger extensor strength correlated very well with the difference in AHA scaled scores (Fig. 2D).

Median dynamometer grip strength values were higher without versus with PEXO (without PEXO: 9.0 kg (IQR: 6.0–10.5 kg), with: 6.5 kg (IQR: 4.0–10.3), Z = − 2.388, *p* = 0.017). Lateral pinch values did not differ (without PEXO: 1.3 kg (IQR: 1.0–2.3 kg), with: 1.5 kg (IQR: 0.8–2.4 kg), Z = − 0.178, *p* = 0.86). Correlations with the difference in AHA scaled score were only fair (Grip strength: ρ = -0.35, *p* = 0.36; lateral pinch strength: ρ = − 0.25, *p* = 0.52).

Patients ID1, 6, 7, 9, and 10 improved in AHA scaled scores more than 7 points (i.e., dichotomous improvement: yes). The results from the ROC analyses show that most measures (MACS, MAS of wrist and fingers, MMT values for shoulder and elbow flexors and wrist extensor, Jamar and lateral pinch dynamometry measurements) could not distinguish significantly between those participants who improved in bimanual performance when wearing PEXO and those who did not. The only measures which could do so were the BBT, SCUES, and MMT of the finger extensors (Fig. 2E–G).

Five out of ten participants (i.e., no data from ID4) could perform a lateral pinch without PEXO, while all participants could perform it with PEXO. Five participants could perform a tip pinch when not wearing PEXO, while seven participants could perform a tip pinch when wearing PEXO (*p* = 0.48). Eight out of ten participants could close the fist without PEXO, but no participant could close the fist when wearing PEXO. For the lateral pinch and fist closure scores, chi-square tests could not be calculated.

Chi-square tests showed a tendency that patients not able to perform a lateral or tip pinch without PEXO improved bimanual performance (as assessed by the AHA scaled scores) when wearing PEXO (for both: Chi-square = 2.723, *p* = 0.099). However, such a tendency was not observed for those who could not initially make a fist (Chi-square = 2.057, *p* = 0.15).

When investigating familiarization with the use of PEXO, participants practiced the pegboard task while wearing PEXO for a median duration of 5 min (IQR: 2.8–8.3 min), varying from 1.5 to 15 min. When evaluating the scores of the pegboard, improvements were noted from pre- to post-practicing with PEXO for the median number of correct placements (pre: 3.0 pegs (IQR: 0.0–6.5); post: 4.5 pegs (IQR: 2.8–11.0), Z = − 2.388, *p* = 0.017). This improvement was not accompanied by a significant change in the time needed to accomplish the task (pre: 120.0 s (IQR: 113.0–120.0); post: 120.0 s (IQR: 77.8–120.0), Z = − 1.826, *p* = 0.062). The post-practice values with PEXO did not differ significantly from the pegboard test performance without wearing PEXO (number of correct placements: 6.5 pegs (IQR: 0.0–11.0), Z = − 0.339, *p* = 0.73; time needed: 119.5 s (IQR: 41.5–120.0), Z = − 0.944, *p* = 0.35).

### Practicability

The median time needed to don the back module (n = 11) amounted to 62 s (IQR: 40–120 s) and varied between 20 and 150 s. No child was able to put the back module on without assistance. The median time required to don the Velcro glove was 150 s (IQR: 30–240 s) and varied between 15 and 430 s. Only ID3 and ID5 were able to put the glove on without help. The median time needed to don the hand module was 160 s (IQR: 120–250 s) and varied between 55 and 340 s.

The technical errors that occurred during the tests are listed in Table 2 and elaborated on in Additional file 1: Technical issues and proposed solutions. The most critical failures were identified to be the robustness and reliability of the transmission system and electronics (issue ID 2.2 and 2.3 with a severity level of 4) and attachment system (issue ID 1.1 and 1.2 with severity levels of 3 and 2, respectively, at frequent occurrence).

**Table 2:** Technical issues

Issue ID	Component	Technical failure mode	Number of occurrences (Subject IDs)	Severity level*
1.1	Attachment system	Subject slips out of the PEXO	3 (1, 6, 11)	3
1.2	Attachment system	Fixation straps loosen	4 (1, 3, 6, 7)	2
2.1	Back module	Transmission cable reached end-stop	1 (8)	3
2.2	Back module	Transmission cable tore	1 (2)	4
2.3	Back module	Booting issues of microcontroller	3 (4, 7, 8)	4
2.4	Back module	Component at the output of the transmission system got loose	2 (5, 8)	1
2.5	Back module	Adjustment of PEXO settings required (reprogramming of microcontroller)	2 (6, 8)	3
2.6	Back module	Button control is not working (control via control unit required)	1 (5)	3
3.1	Hand module	Screw transmitting force to the little finger loosened, reducing movement of the little finger	1 (10)	2
3.2	Hand module	Thumb position of PEXO not fitting the subject (too proximal)	2 (6, 10)	2
3.3	Hand module	PEXO cover on back of the hand loosened	1 (10)	2

*Severity levels: 1 Negligible issue not influencing performance or functionality, 2 Marginal issue allowing successful task completion, but (i) leading to a small delay (< 1 min) and/or (ii) requiring additional action/adjustment by the user, 3 Issue requiring allowing successful task completion, but (i) leading to a major delay (> 1 min) and/or (ii) requiring support from a caregiver, 4 Critical issue (i) requiring intervention by the study coordinator to avoid potential harm to the participants and/or (ii) preventing task completion due to total failure of the device requiring technical maintenance

### Acceptability

The responses of the participants and the occupational therapist are shown in Table 3. Responses varied considerably between participants and questions. Interestingly, for the questions P1 to P5, we found good to very good correlations between the subjective impressions of the participants and the difference in AHA scaled scores (with minus without PEXO conditions). There was only a fair relationship for question P6.

**Table 3:** Acceptability of PEXO by participants and therapist: custom-made questionnaire

Responder	Questions	Number of responses					Median (IQR)	Correlation with AHA
		1	2	3	4	5		ρ (p-value)
Participants	P1. I found PEXO comfortable to wear			3	3	4	4.0 (3.0–5.0)	0.63 (0.069)
	P2. I found training with PEXO interesting	1	1			8	5.0 (4.3–5.0)	0.64 (0.062)
	P3. The tasks were easier to perform with PEXO.*	5		1		3	1.0 (1.0–5.0)	0.67 (0.070)
	P4. With PEXO, I have better control over my hand activities.*	4		1		3	2.0 (1.0–5.0)	0.86 (0.014)
	P5. I would like to continue training with PEXO	3			2	5	4.5 (1.0–5.0)	0.87 (0.002)
	P6. PEXO could be put on quickly	1	2	4	1	2	3.0 (2.0–4.3)	0.30 (0.43)
Therapist	T1. Putting on the PEXO was easy	1	3	2	3	1	3.0 (2.0–4.0)	-0.56 (0.119)
	T2. The child did not need any external motivation during the whole session	2				8	5.0 (4.0–5.0)	0.05 (0.894)
	T3. The child could carry out goal-oriented training with PEXO	3		2		5	4.0 (1.0–5.0)	0.69 (0.039)
	T4. The tasks with PEXO were easy to complete		2	4	3	1	3.0 (2.8–4.0)	-0.61 (0.079)
	T5. Using the button was easy			2	1	7	5.0 (3.8–5.0)	-0.59 (0.094)

Responses varied from 1 (not at all) to 5 (very much). Please note, the questions were translated from the German language. * Incomplete responses: ID6 did not understand question 4, ID7 did not understand questions 3 and 4. Correlations were performed between the ratings and the difference in AHA scaled scores between the with-minus-without PEXO conditions. Abbreviations: IQR, inter-quartile range; ρ, Spearman’s correlation Coefficient

Concerning the open questions (Table 4), participants appreciated that they could do more with the hand when wearing PEXO (ID1), opening and closing the hand with pressing a button was possible (ID3), one could play with it (ID5), it was fun (ID8), and the hand felt alive again and its function improved (ID10).

**Table 4:** Summary of answers to open questions by participants

What did you like about the training with PEXO?		What did you dislike about the training with PEXO?	
Participant ID	Answer	Participant ID	Answer
1	PEXO increases my hand function	1	It takes patience until PEXO is donned. It should be softer
3	I can open and close PEXO by pressing the button	2	It is hot inside the glove
4	It is a cool feeling	3	I can not move my fingers independently
5	I can play with it	4	I couldn’t try it with my weaker hand.*
8	It is funny	5	It is hot inside the glove. The backpack left pressure marks on my shoulder, and it got heavy over time
10	My hand felt alive again and its function improved	7	I was sweating in the glove
		8	I was sweating in the glove. The glove was a bit tight but didn’t hurt

All answers were translated from German. *Hand module was not available due to malfunction

One participant (ID1) disliked that she needed patience while donning PEXO and mentioned that it should be softer. Many participants commented that wearing the glove resulted in a warm and sweaty hand (ID2, 5, 7, and 8). Furthermore, participants mentioned that individual movements of the fingers were not possible (ID3) and that the back module became heavy over time and pressed on the shoulder (ID5).

Five patients reported the sensation of pressure on the skin. VAS scores indicating the level of pain were very low (0–2) for four patients. ID5 reported pain at the dorsum of the hand (VAS 7.5) and the little finger and thumb (VAS 5). No other PEXO-related adverse events were noted.

We asked the participants whether they could think of activities that they could perform with PEXO. They mentioned activities such as closing (the zipper of) a jacket (ID1 and 9); holding, opening, or drinking from a bottle (ID1, 3, 7); opening crayons or lip-gloss or pushing a shopping cart (ID1); tying shoes (ID3, 9 and 10); brushing teeth (ID3, 8, 9); holding a knife, cutting food or eating with cutlery (ID7, 8, 9, 10); holding a book or a mobile phone, opening a door or playing videogames (ID8); or holding a sharpener (ID10).

The responses of the therapist varied widely between participants (Table 3). The responses to statement T3 showed that the opinion of the therapist which patient could perform a goal-oriented training with PEXO correlated well with the differences in AHA scaled scores, which were calculated a-posteriori, i.e., these responses were not available at the time of responding. The correlations with the other questions were negative and of moderate sizes only.

The therapist responded further in the open questions that PEXO would be better adjustable for several children if the wrist of PEXO could be flexible. For children with wrist and finger contractures, a flexible wrist joint would make it easier to don PEXO. Generally, a flexible wrist joint could make grasping movements more physiological.

## Discussion

While others have developed soft hand exoskeletons to support thumb [10] or finger [11] motion in children, this study presents the first evaluation of the appropriateness, practicability, and acceptability of a whole hand wearable exoskeleton in a pediatric user group. We consider such technology essential, particularly as children are a very vulnerable group. This technology, when used at a critical phase of a child’s development, could improve both the quality of life and the long-term health prospects [9]. Furthermore, adult patients with stroke participating in a similar trial frequently reported that the exoskeleton should become available in smaller sizes to fit small hands [34]. This shows the need for reducing the size of such technologies to include more patients.

Concerning the appropriateness results, we identified those children with upper limb impairments that could improve bimanual performance while using PEXO, i.e., those with low BBT, SCUES, and finger extensor values. Results from the Smart Pegboard showed that the participants needed only a short practice period to improve the handling with PEXO. Concerning the practicability, it was noted that most children needed help with donning. Furthermore, we identified and rated any technical issue and feedback received from participants and the therapist towards the design and robustness of PEXO. Interestingly, the participants' subjective acceptance of PEXO and the impression of the therapist whether a particular child could perform a goal-oriented therapy with PEXO correlated well with the objective improvement in bimanual performance caused by PEXO. By combining the insights regarding these dimensions of clinical utility, we formulated recommendations to improve PEXO and paved the way for its successful clinical application.

### Identifying participants who could benefit from PEXO

This study showed that patients with poor upper limb function could benefit from a wearable soft exoskeleton. This is in line with results from adults with spinal cord injury, where participants with lower baseline motor function received significant benefits from soft robotic glove assistance [35], and participants with loss of hand function improved while wearing a soft robotic glove [36]. However, an overall classification instrument like the MACS did not assess impairments in sufficient detail to select patients who might benefit from using PEXO. The most promising classifiers seem to be the BBT as a simple, practical, and reliable measure of gross manual dexterity, the SCUES, a measure quantifying selective voluntary motor control, and the manual muscle testing of the finger extensors. Indeed, lack of finger extension was one of the inclusion criteria in the study of Yurkewich and colleagues [34], and these adult patients with stroke showed considerable improvements in hand use when wearing the Hand Extension Robot Orthosis (HERO) Grip Glove compared to no exoskeleton.

While the results of our ROC analyses propose specific cut-off values to identify patients who might be suitable for training with PEXO, we emphasize that these numbers should be interpreted cautiously due to the small number of participants that were involved. At the current stage, we recommend that more extensive trials are needed to investigate whether these assessments prove valuable in selecting appropriate patients for training with wearable exoskeletons like PEXO.

In patients with better hand and arm function, PEXO seemed to slow down the movements (e.g., Fig. 2B) due to the required coordination between positioning the grasping hand and the hand triggering PEXO by pressing the button, and also the time PEXO needs to close. Similar results were observed in studies with adult users. Correia and colleagues reported that the button control was an effective intention detection method [35]. They found that higher-functioning adult patients with spinal cord injury were also slowed down in tasks that they could perform without wearing the glove, whereas lower-functioning individuals were challenged by engaging both limbs simultaneously to hold an object with one hand and press a button with the other [35]. Yurkewich et al. [34] reported that hand exoskeletons controlled by a button or an automatic mode based on inertial measurement unit data decreased BBT scores in adults with higher baseline scores. The number of blocks transferred within one minute was in a comparable range to the results obtained in our study with button control (mean 2.9 blocks in [34]). Participants in [34] preferred the automatic mode, performing slightly better than button control (3.3 blocks). Particularly in children and adolescents, a technology that might slow them down is unlikely to receive high acceptance. Researchers developing assistive technologies for children should consider putting additional effort into designing control modalities that are robust, intuitive, and easy to use by children. We plan for our subsequent evaluations to investigate the use of several control systems: (i) a myoelectric control system (e.g., [37, 38]), (ii) a sensor glove that embeds the user input directly into the movement through contact detection with the object to grasp [39], and (iii) voice control (e.g., [40]). Such control systems might be more intuitive, speed up the control of the hand, and be beneficial, particularly for bimanual tasks.

The range of motion supported by PEXO is not sufficient to fully close the hand to a fist. This could partly explain why we found no differences during dynamometer testing where almost full closure is needed to exert pressure on the dynamometer. Resultingly, PEXO could not sufficiently assist the subjects in the grip dynamometer task. The selection of patients could also explain this finding. Several participants could close a fist without wearing PEXO, but the numbers of those who could not do so were small, which might have affected the statistical power. Nevertheless, it seems to be a limitation of the current prototype, and increasing the range of motion and force production would be desirable. A full hand closure is not critical for most daily-life relevant tasks as patients would use PEXO to hold objects that do not require a complete closure of the hand nor maximal grip strength. Overall, our data shows that in children and asolescents, bimanual hand use increased when wearing PEXO.

Interestingly, even though dynamometer assessments are highly reliable, they did not seem good estimators for identifying patients that could improve bimanual performance with PEXO, unlike, for example, the MMT of the finger extensors. This difference can have several causes. For example, it is known that patients with neurological lesions frequently have more difficulties in opening the hand (i.e., extending the fingers) than in closing. Furthermore, active finger extension has previously been identified as a predictor of functional improvement in adult patients with stroke [41–43]. Another explanation could be the difference in rating. While the MMT takes the range of motion into account, we did not individualize the dynamometer values (for example, by normalizing them for sex, age, or anthropometrics).

### Familiarization with the use of PEXO

Immediate improvements in grasping function has been observed in several studies. This was seen in an adult individual using the RELab tenoexo after suffering a spinal cord injury [13], in adults with spinal cord injury wearing a textile-based soft robotic glove [35, 36], and in adults after stroke wearing the HERO Grip Glove [34]. While we also noted immediate improvements in certain tests, the performance of children with some hand function worsened in tests involving a timed component when using PEXO. For example, the Smart Pegboard task showed that the number of correctly inserted pegs improved after the practice period, while the time remained the same. We assume that patients could control increased precision in their hand without necessarily getting faster. Certain participants got faster in subsequent trials, but were still unable to complete the task of inserting all 11 pegs in under 120 s. Indeed, several relevant aspects of the task might require some time to get used to, for example, opening and closing PEXO using the pushbutton or the correct positioning of the lower arm and wrist so that by closing PEXO, the pegs can be grasped. Overall, the participants required a median practice time of around 5 min to improve the performance with the hand exoskeleton. This duration seems acceptable and lies in the range of training times reported in adults after stroke [34, 44]. However, the practice time with the Smart Pegboard varied largely between participants (from 1.5 to 15 min). While we had initially planned 20 min to practice hand opening and closing with PEXO, we noticed that some children learned this task within minutes. To avoid losing motivation and compliance due to too many practice sessions, the therapist decided to continue with the protocol on an individual basis, i.e., if the child could perform basic grasping tasks with PEXO. The participants had very diverse motor and cognitive impairments and were at different stages of motor development, which influenced the time needed to learn new tasks differently in each child. We expect that a more intuitive control system will speed up the performance of tasks and will contribute to an even higher acceptance level in participants.

### Practicability and suggestions for improvement

The donning process is critical to ensure unrestricted use of assistive devices. In this study, participants could not don all of the components independently, which falls in line with other studies performed in adult patients with spinal cord injury (e.g., [35]) or stroke (e.g., [34]). In the latter study, participants reported the lowest satisfaction scores for ease of donning. While help with donning is not limited during one-to-one therapy sessions, the individual using PEXO depends on assistance from another person in daily life situations. The participants of this cross-sectional study were exposed to the technology for the first time, and we expect that participants can increase donning capabilities after practicing. Further, increasing the adaptability of the assistive device to individual needs will not only benefit the donning process but also improve the functionality and wearing comfort. Some participants experienced pressure points, and the thumb position did not fit perfectly. Modular and adaptable designs for children’s hand exoskeletons are needed to account for the fact that young patients will grow, and the devices need to be adjusted to the changing anthropometrics over time.

PEXO currently allows no movement in the wrist joint. The therapist commented that a flexible wrist could simplify donning and doffing and make grasp movements more physiological. Indeed, Valevicius and colleagues showed that in healthy adults performing a cup transfer task that included reach, grasp, transport, and release phases, wrist flexion/extension varied significantly [45]. For example, when the participants grasped the cup from the top at the rim and moved the cup, the wrist showed a mean peak flexion angle of 45° combined with a peak ulnar deviation angle of 28°. However, when moving the cup while holding it from the side, the wrist showed a mean peak extension angle of 33° and a radial deviation of 9°. This example underlines that wrist movements play an important role in specific tasks, and restricting movement of that joint will affect the kinematics of the other upper limb joints (e.g., leading to compensatory movements). However, as children with CP show different trunk and upper limb kinematics during reach-to-grasp movements compared to typically developing children (i.e., increased trunk movements, reduced shoulder elevation, elbow extension, and supination, and increased wrist flexion) [46], further research is needed to verify whether adding wrist flexion will make multijoint movements more physiological in the target group.

Based on the technical issues that occurred during the sessions and the comments from the participants and the therapist, we identified critical components that need to be improved. This includes the transmission system and attachment of the hand exoskeleton with the glove. Accordingly, we formulated recommendations on how these could be resolved (Additional file 1: Technical issues and proposed solutions). We will investigate the clinical utility of several of these improvements in future studies.

### Acceptance by participants and therapist

It is interesting to see that the participants’ subjective impression of acceptability of the technology correlates well with their improvements in bimanual performance. Statements such as 'With PEXO, I have better control over my hand activities' or 'I would like to continue training with PEXO' are meaningful indicators of the potential of such technologies. Also, the therapist's expert opinion on whether 'The child could carry out a goal-oriented training with PEXO' could be validated by objective data.

The most commonly mentioned 'dislike' was that the glove resulted in a warm, sweaty hand. Similar concerns were highlighted in studies with adults employing a full-hand soft robotic glove [44]. If participants are expected to wear such technologies for extended periods of time, designers need to consider these comments early during their development stage. Otherwise, the comfort of wearing PEXO seemed sufficient based on the participants’ feedback in the custom-made questionnaire. While the young patients reported several daily life activities that they could perform with PEXO, realistically, not all of these activities can be performed with the current version of PEXO (e.g., tasks that require high dexterity such as tying shoes or playing video games).

Some participants reported pressure of the glove and exoskeleton at specific locations. Pressure might result from incorrect sizing of PEXO for children, highlighting the need for tailored exoskeletons fitting the specific hand size for optimal adoption of such technologies. However, no skin lesions were observed, and the pain levels reported for these locations were generally low (≤ 2/10). Only one participant reported higher VAS values at locations where pressure was perceived (7,5 and 5 from 10). However, at the same time, this patient rated the comfort of wearing PEXO as good (4/5). This highlights the challenges when evaluating such technologies in children and adolescents.

### Methodological considerations

The therapist in this study was involved in the testing and, therefore, not blinded for the clinical assessments and the participants’ responses. One could argue that this would have influenced some correlations, particularly those of the AHA scaled scores. However, the AHA scores and analysis were performed later after the test session from video recordings. Furthermore, the occupational therapist was initially unaware of the details of the planned data analyses. Therefore, the lack of blinding did not result in a large bias.

Despite our sample being already quite heterogeneous, all participants had low spasticity scores for the wrist or fingers. Therefore, we cannot conclude from this study whether PEXO could provide sufficient force to overcome higher spasticity levels. Besides, as the proximal muscles were relatively strong, we do not know whether children or adolescents with weaker proximal muscles could benefit from PEXO to improve bimanual performance.

The kids-AHA has been developed and validated for children between 18 months and 12 years with unilateral CP or plexus brachialis lesion. However, it was applied to all children and adolescents in this study to improve comparability between the tasks and scorings.

We recruited several children who wore PEXO sizes 2 or 3, one adolescent who wore the adult tenoexo, and one participant who wore the smallest version. Recruiting children who could wear the smallest version was more complicated because we only had a right-hand version. Nevertheless, we did not find any indication that the uneven representation of PEXO sizes might have affected our findings.

## Conclusion

PEXO is a safe, wearable soft exoskeleton developed for children and adolescents with minimal hand function. PEXO can improve bimanual hand performance and enable the lateral and tip pinch in participants who otherwise could not perform these tasks independently. We identified several factors (dexterity, arm and hand selective motor control, and finger extensor strength) that, in the long-term, might prove meaningful in identifying patients who might be suitable for training with or using a wearable exoskeleton for the hand. PEXO was well-accepted by patients, who can increase their bimanual performance when using it. The subjective opinion of the patient and an experienced therapist after a 2-h training seem additional good indicators of whether PEXO can improve functional abilities. The current PEXO prototype has several design shortcomings that need to be addressed before being tested under less standardized, more realistic daily-life conditions as an assistive device. Still, this study proved the feasibility of PEXO regarding its clinical application and highlighted its potential benefit for children with severe upper limb impairments.

## Supplementary Information

**Additional file 1:** Technical issues and proposed solutions.

## Data Availability

The datasets generated and/or analyzed during the current study are not publicly available.

## Abbreviations

AHA: Assisting Hand Assessment
AUC: Area Under the Curve
BBT: Box and Block Test
FMECA: Failure mode, effect, and criticality analysis
HAT: Hypertonia Assessment Tool
HERO: Hand Extension Robot Orthosis
ICF-CY: International Classification of Functioning, Disability and Health – Children and Youth version
IQR: Inter-quartile range
MAS: Modified Ashworth Scale
MMT: Manual Muscle Testing
PEXO: Pediatric hand exoskeleton
ROC: Receiver Operating Characteristics
SCUES: Selective Control of the Upper Limb Scale
Sens.: Sensitivity
Spec.: Specificity
VAS: Visual Analogue Scale
WeeFIM: Functional Independence Measure for Children
