# Supplementary material for: Clinical utility of a pediatric hand exoskeleton: identifying users, practicability, and acceptance, and recommendations for design improvement
Source: J Neuroeng Rehabil. 2022 Feb 11;19:17. doi: 10.1186/s12984-022-00994-9 (PMC8832660; doi:10.1186/s12984-022-00994-9)
Supplement: Supplementary file 1 — Additional file 1: Technical issues and proposed solutions. [file 12984_2022_994_MOESM1_ESM.docx]

# Appendix 1: Technical issues and proposed solutions

## Attachment system

Issue 1.1:
Several subjects mentioned that the glove used to don the hand exoskeleton leads to sweating and discomfort. Furthermore, subject ID1, ID6, and ID 11 slipped out of the glove towards the end of the session leading to decreased support by PEXO and suboptimal grasping patterns.

Solution 1.1:
The glove design needs to be optimized, e.g., featuring an open palm to reduce sweating. An open-palm design would further allow for additional sensory feedback, which is restricted with the current glove. Slipping out of the glove could be avoided by better fixing the glove on the proximal side, e.g., around the wrist.

Issue 1.2:
The straps fixing the hand exoskeleton opened or got loose for subjects ID1, ID3, ID6, and ID7, resulting in a loose fit of the hand exoskeleton on the hand and decreased force transmission.

Solution 1.2:
The hooks fixing the straps need to have a tighter fit or could be avoided by directly fixing the straps to the hand module.

Back module

Issue 2.1:
Before the test with subject ID8, the transmission cable of the actuation system reached the end stop of the winding on the pulley and had to be manually adjusted, leading to a delay.

Solution 2.1:
The position of the cable system would need to be tracked, e.g., using encoders on the motor.

Issue 2.2:
In the test with subject ID2, the transmission cable of the actuation system driving the thumb of the hand exoskeleton tore. In consequence, the test could not be completed.

Solution 2.2:
The durability and strength of the remote actuation system need to be improved by, e.g., evaluating alternative Bowden cables.

Issue 2.3:
Issues occurred during the start-up process of the electronics (ID4, ID7, and ID8), leading to a delay in the therapy session.

Solution 2.3:
The problem source was identified in the wiring in the backpack and the robustness of the used microcontroller. Improving the wiring and considering other microcontrollers are the consequent solutions.

Issue 2.4:
A small plate on the actuation unit of the thumb, fixing the rack, got loose in the tests with subjects ID5 and ID8.

Solution 2.4:
The plate is currently glued to the thumb actuation. Designing an attachment by form fit or relying on screws might solve the issue.

Issue 2.5:
The settings of PEXO (e.g., maximum force, closing time) had to be adjusted in the code via laptop in subject ID6 and ID8. This requires depowering and rebooting the system, leading to a delay.

Solution 2.5:
The time required to adjust these settings could be reduced by allowing users or therapists to access the settings via a mobile app or a similar input device connected to the microcontroller (e.g., cable, Bluetooth).

Issue 2.6:
For subject ID5, the control of the hand exoskeleton via button did not work.

Solution 2.6:
The problem source could be identified to be the wiring in the back module. Improving the wiring is consequent solutions.

## Hand module

Issue 3.1:
A screw that is part of the force transmission chain within the hand module (between push spring and sliding spring) got loose, reducing the movement of the finger mechanism, especially when opening. During the closing, the force can still be transmitted. When opening, the leaf springs of the finger mechanism pull back to a straight position but to fully open a user’s hand, this screw is required to transmit the force from the actuators.

Solution 3.1:
The screw could be secured using Loctite adhesives or similar.

Issue 3.2:
The thumb position did not fit the subject's hand (ID6, ID10).

Solution 3.2:
For this feasibility study, PEXO hand modules were prepared in three different sizes based on anthropometric data. Therefore, it needed to be expected that the fit to varying hand sizes of children of different age and neurological conditions could be challenging.
However, a tailor algorithm implemented in the CAD model of the hand exoskeleton could be used to adapt the hand module to a specific user.

Issue 3.3:
The wrist cover of the hand module, to which the thumb is attached, lifted off (ID10).

Solution 3.3:
The wrist cover is only clipped to the hand module. The attachment could be improved (e.g., fixing with screws).

Issue 3.4:
Five patients reported uncomfortable pressure sensation on the skin.

Solution 3.4:
See solution 3.2.

Issue 3.5:
In some subjects, the therapist observed compensatory trunk movements during grasping tasks.

Solution 3.5:
An additional degree of freedom in the hand module allowing wrist flexion and extension could potentially reduce compensatory movements compared to the currently fixed wrist design.
